# Linking Bovine Tuberculosis on Cattle Farms to White-Tailed Deer and Environmental Variables Using Bayesian Hierarchical Analysis

**DOI:** 10.1371/journal.pone.0090925

**Published:** 2014-03-03

**Authors:** W. David Walter, Rick Smith, Mike Vanderklok, Kurt C. VerCauteren

**Affiliations:** 1 U.S. Geological Survey, Pennsylvania Cooperative Fish and Wildlife Research Unit, Pennsylvania State University, University Park, Pennsylvania, United States of America; 2 Animal Industry Division, Michigan Department of Agriculture and Rural Development, Lansing, Michigan, United States of America; 3 United States Department of Agriculture, Animal and Plant Health Inspection Services, Wildlife Services, National Wildlife Research Center, Fort Collins, Colorado, United States of America; National Institute for Agriculture and Veterinary Research, IP (INIAV, I.P.), Portugal

## Abstract

Bovine tuberculosis is a bacterial disease caused by *Mycobacterium bovis* in livestock and wildlife with hosts that include Eurasian badgers (*Meles meles*), brushtail possum (*Trichosurus vulpecula*), and white-tailed deer (*Odocoileus virginianus*). Risk-assessment efforts in Michigan have been initiated on farms to minimize interactions of cattle with wildlife hosts but research on *M. bovis* on cattle farms has not investigated the spatial context of disease epidemiology. To incorporate spatially explicit data, initial likelihood of infection probabilities for cattle farms tested for *M. bovis*, prevalence of *M. bovis* in white-tailed deer, deer density, and environmental variables for each farm were modeled in a Bayesian hierarchical framework. We used geo-referenced locations of 762 cattle farms that have been tested for *M. bovis*, white-tailed deer prevalence, and several environmental variables that may lead to long-term survival and viability of *M. bovis* on farms and surrounding habitats (i.e., soil type, habitat type). Bayesian hierarchical analyses identified deer prevalence and proportion of sandy soil within our sampling grid as the most supported model. Analysis of cattle farms tested for *M. bovis* identified that for every 1% increase in sandy soil resulted in an increase in odds of infection by 4%. Our analysis revealed that the influence of prevalence of *M. bovis* in white-tailed deer was still a concern even after considerable efforts to prevent cattle interactions with white-tailed deer through on-farm mitigation and reduction in the deer population. Cattle farms test positive for *M. bovis* annually in our study area suggesting that the potential for an environmental source either on farms or in the surrounding landscape may contributing to new or re-infections with *M. bovis*. Our research provides an initial assessment of potential environmental factors that could be incorporated into additional modeling efforts as more knowledge of deer herd factors and cattle farm prevalence is documented.

## Introduction

Bovine tuberculosis (bTB) is a bacterial disease (*Mycobacterium bovis*) in livestock and wildlife that results in United States Department of Agriculture-mandated depopulation of cattle herds costing farmers millions in lost revenue throughout the world [1], [2]. Preliminary efforts by the Michigan Department of Agriculture-Animal Industry Division (MDA) have created protocols that farmers could follow to reduce potential for *M. bovis* infection of cattle in Michigan’s Modified Accredited Zone (MAZ) [3]. Basic risk-assessment efforts were needed, however, to address the spatial context of disease epidemiology (i.e., infection probability if a farm is adjacent to a bTB-infected farm) and dynamics of primary reservoirs in the MAZ (i.e., white-tailed deer [*Odocoileus virginianus*]).

The influence of wildlife activity on transmission of *M. bovis* depends on possible hosts and their ability to transmit disease [4]–[6]. Direct observation of farms in Michigan, USA documented that indirect interactions between cattle and white-tailed deer were dominated by use of pastures and silage storage areas but deer fed from hay racks or silage troughs on only one occasion [7]. Visitation of farm yards and cattle-use areas by sixteen GPS-collared white-tailed deer was documented in Michigan’s MAZ and deer were documented using confined feeding areas, water tubs, and pastures [8]. Prevalence of *M. bovis* in deer was as high as 10–12% in some townships but currently can range from 2 to ≥5% in some townships due to changes in management regulation for deer and feeding on some cattle farms [3], [9], [10].

Reoccurrence of *M. bovis* in farms depopulated of cattle in Michigan would suggest an environmental or mammalian host source of re-infection as several farms have become re-infected with *M. bovis* on ≥2 separate occasions often spanning 3–7 years between re-infection [3], [11]. Under natural shaded conditions on pastures, survival of *M. bovis* in cattle feces was documented to span up to 5 months post-application during winter but only up to 2 months during spring and summer [12]. Effluent plots tested positive for *M. bovis* for up to 29, 13, and 35 days post application for soil, radishes, and lettuce, respectively, in a study in raised garden plots (lined plywood boxes) [13]. Although environmental and anthropogenic variables that influence odds of contracting a disease have been addressed in North America [3], [14], only recently has the spatial matrices incorporating proximity to adjacent infected individuals been successfully modeled in disease epidemiology research with advances in software (i.e., WinBUGS; [15]–[17]). Understanding the spatial dynamics of *M. bovis* will increase our ability to predict future spread or occurrences and variables influencing these occurrences across the MAZ in the northern, lower peninsula of Michigan.

To incorporate spatially explicit data, likelihood of infection probabilities within a geographically designed grid can be determined for cattle herds that tested positive for *M. bovis* and incorporated into a Bayesian hierarchical framework. Although on-farm management practices are believed to influence *M. bovis* transmission, consensus on the most important farm-level factor responsible for transmission is absent and varied across studies in Europe and North America ([11], [18], [19] but see [3] for a detailed summary). Spatially explicit data on environments that cattle farms occupy is often lacking for researchers attempting to understand the underlying distribution of disease in the landscape and has not been modeled in this system since discovery of *M. bovis* in a free-ranging white-tailed deer in 1975. Our objectives were to model odds of infection with *M. bovis* in cattle farms at the herd level using Bayesian hierarchical analysis by incorporating prevalence of *M. bovis* in the deer population, environmental variables, spatial structure, and unstructured spatial heterogeneity across the MAZ in Michigan. An understanding of conditions that sustain survival of *M. bovis* in the environment would be valuable to our ability to focus surveillance for the disease and predict future spread or occurrences outside of the MAZ in Michigan.

## Materials and Methods

### Study area

**Figure 1:**
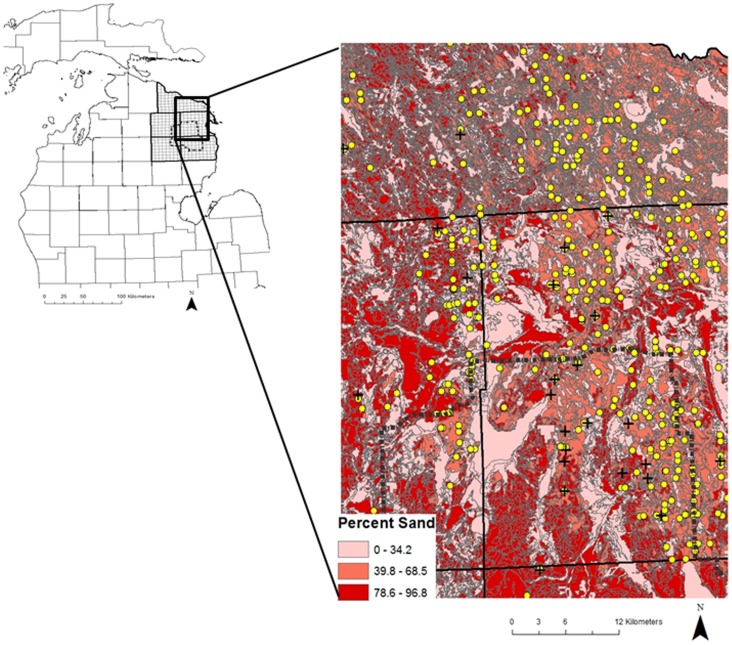
Sampling grid (25 km^2^ cells) that contained all cattle farms tested for bovine tuberculosis within the Modified Accredited Zone (5 counties in bold) in the upper, lower peninsula of Michigan. Deer Management Unit 452 (dashed polygon) is considered the bovine tuberculosis core area for surveillance in white-tailed deer (Outset). Cattle farms that tested negative (yellow circles) and positive (black crosses) for *Mycobacterium bovis* used in Bayesian Hierarchical models overlayed on percent sand within a portion of the study area in the upper, lower peninsula of Michigan (Inset).

We conducted our study in the northern, lower peninsula of Michigan in the MAZ. The 8,062 km^2^ study area included the entirety of Alcona, Alpena, Montmorency, Oscoda, and Presque Isle counties (Fig. 1). The area encompassed the majority of the cattle farms where *M. bovis* has been found in Michigan. Our study area surrounds Deer Management Unit 452 that has been defined as the bovine tuberculosis core area by the Michigan Department of Natural Resources (MDNR) due to the high prevalence of *M. bovis* in free-ranging deer and the presence of *M. bovis*-positive cattle on farms (Fig. 1; [20], [21]). Vegetation categories present in our study area included: *developed* that included roads, development, and barren land; *grass* that included pasture/hay fields and native grasses; *agriculture* that included crops other than forage; *forest* that included upland hardwood stands (*Quercus alba*, *Acer rubrum*, and *A. saccharum*), aspen stands (*Populus tremuloides* and *P. grandidentata*), hardwood/aspen mixed stands, upland conifer stands (*Pinus glauca*, *P. banksiana*, and *P. resinosa*), and hardwood/conifer mixed stands, and swamp that included lowland conifer forests/swamps (*P. glauca*, *P. mariana*, *Thuja occidentalis*, *Abies balsamea*. and *Latrix laricinea*). Elevations in the area ranged from 150–390 m above sea level and the mean annual temperature was 6.6°C, the mean rainfall was 72.5 cm, and there was a mean snowfall of 175 cm [22].

To link the disease status (positive or negative) of each farm in the sample to deer herd and environmental-level predictors, we first overlaid a 5×5 km square grid having a resolution of 25 square kilometers (hereafter referred to as *grid cell*), which is equal to a quarter township in size. We selected quarter townships as the proper resolution given that township would likely be too coarse a scale and section would be too fine a resolution for model convergence based on previous research with Bayesian hierarchical models [17]. There were a total of 368 grid cells covering the MAZ and we assigned each farm in our study to its appropriate grid cell.

### Observation component

#### Cattle farm data

Our data included 762 cattle farms of known infection status (*observation component*) provided by the Michigan Department of Agriculture and Rural Development (MDA) from mandatory testing of cattle on an annual basis. Based on current knowledge that indirect transmission (i.e., environmental source) of *M. bovis* to cattle may be important, we used replicates for each farm that tested positive on ≥1 occasion over the 11 year span of our study (58 positive, 704 negative) with positive farms coded as 1 and negative farms coded as 0. We included a farm each time it tested positive for *M. bovis* and this was deemed warranted because conditions of that farm or host characteristics in the area were responsible for continued infections of *M. bovis* and replicates would weigh environmental characteristics of farms that tested positive on >1 occasion. Because farms that tested positive for *M. bovis* were depopulated of cattle then re-populated prior to subsequently testing positive, each farm was considered an independent observation for the purposes of our study design. Each farm was tested annually for *M. bovis* but we did not include additional negatives as replicates because that would have likely masked the effects of the positives that we were attempting to model for odds of infection.

We included all cattle farms in this region because we wanted to determine the environmental drivers of disease that were not associated with farm practices and remained unaltered when cattle farms were depopulated or permanently closed (e.g., surrounding habitats, soil composition). Furthermore, we did not include any farm-level covariates (e.g., herd size, feeding practices) in our models because farm mitigation strategies were initiated by the MDA during our study [3], would be difficult to quantify and standardize across the study region, and would only be considered a contamination source (e.g., cattle fed in deer habitat) but would not influence environmental persistence or survival of *M. bovis* in the landscape.

### Process component

#### Host-level variables

The MDNR provided section-level data on deer prevalence for *M. bovis* from 1995 to 2009. We limited our analysis to white-tailed deer prevalence for 2005 to 2009 because deer herd management, ban on baiting deer, and mitigation of on-farm practices indicated that deer prevalence has stabilized within the past 5 years thus, more reflective of current deer prevalence [10], [23]. Apparent prevalence of *M. bovis* in deer was determined for each grid cell by dividing the total number of deer testing positive by the total number of deer tested resulting in percent prevalence that was entered into models. Annual deer densities were provided by the MDNR from 2005 to 2009 at the county level for the MAZ based on Sex-Age-Kill reconstruction technique or additional methods if available [24], [25]. We averaged deer density over the time period to match deer prevalence (2005–2009) that resulted in a single estimate of deer density per grid cell. Due to the logistical constraints of estimating deer densities at a fine scale, such as to the section-level, we used the only available data to represent deer densities in our study site as deer per square kilometer. We did not select mean deer prevalence or mean deer density for the entire span of sampling of cattle farms because we were interested in modeling the effects of more recent deer prevalence that likely would be influenced by initiation of deer and on-farm management practices in 1996.

#### Environmental-level variables

We hypothesized *a priori* that *M. bovis* patterns on farms were structured in part by spatial heterogeneities in features of the landscape, therefore, we identified four environmental-level predictors of infection based on optimal survival characteristics of *M. bovis* identified in the literature [3]. Proportion of sand content in the soil was selected because dry sandy loam soils at the proper pH and moisture promoted bacteria growth [26], [27]. Proportion of the landscape ponding frequently and proportion of swamp/wetland were selected because the duration of standing water occurring in non-wetlands (ponding) and soil characteristics of inundated areas (wetlands) were conducive to long-term survival of *M. bovis* [27], [28]. Mean soil pH was selected because soil pH from 5.8 to 6.9 was conducive to culture of *M. bovis* at the optimum temperature (37°C) for survival [27], [29]. Sand (where “sand” was defined as soil particles with size >2 μm), landscape ponding frequently, and soil pH was determined using the Advanced Mode of the Soil Data Viewer available through the National Resources Conservation Service of the US Department of Agriculture in ArcMap 9.x (ArcMap; Environmental Systems Research Institute, Redlands, CA, USA). Soil Data Viewer provides interactive mapping software to query the Soil Survey Geographic (SSURGO) database and descriptive characteristics for each soil type. Sand was expressed as the percent sand within a soil type polygon (∼2 ha resolution) for each soil map unit [30]. Each grid cell was thus potentially composed of multiple soil type polygons with varying sand contents. Therefore, we calculated the mean percent sand for each 25 km^2^ grid cell using a weighted average based on the area of the various soil type polygons and their associated sand content. Similarly, in the Advanced Mode of the Soil Data Viewer, we identified the proportion of each grid cell that ponded frequently with frequently defined as “ponding occurs, on the average, more than once in 2 years and the chance of ponding is >50% in any year [30].” Similar to sand and ponding frequently, we calculated the mean soil pH for each 25 km^2^ grid cell using a weighted average based on the area of the various pH polygons and their associated pH value.

We used the National Land Cover Database of 2006 (NLCD) that was created from Landsat 7 imagery to determine the proportion of swamp/wetland across the study site (MRLC 2007). To standardize analyses across the MAZ, we reclassified land cover from the NLCD into 8 categories used in Kaneene et al. [11]: hardwood forest, coniferous forest, mixed forest, open areas and shrubs, wetland/swamp, agricultural use, open water, and other (industrial, residential). We extracted the proportion of wetland/swamp from NLCD within each grid cell with all environmental-level variables, except soil pH, presented as a percentage in a grid cell in modeling efforts. Skewness of data and correlation among covariates was assessed but data transformations and exclusions were not considered necessary prior to entering into models.

### Statistical analysis

We used a Bayesian hierarchical model structure [31], [32] with logistic regression models (described below) to examine how recent TB prevalence at the deer herd-level and landscape factors influenced the probability of a farm being infected, while adjusting for the other covariates and spatial structure in the data [33]. To adjust for latent spatial effects we included two types of random effects that captured both the influence of the local neighborhood (i.e., cells sharing a border or vertex with each 25-km^2^ grid cell; CAR) in determining spatial clustering of *M. bovis*, as well as any spatially independent influences occurring at the 25-km^2^ spatial resolution of our grid (HET). Our models were constructed hierarchically to accommodate the fact that information from multiple levels (i.e., fixed-effects and spatial random effects) was being used to estimate individual-level infection probabilities. Taking a Bayesian approach, we used Markov Chain Monte Carlo (MCMC) simulation methods available within the program WinBUGS [34] to produce the unnormalized joint posterior density for the parameters of interest across all models examined based on the product of the data likelihood and the prior densities for each parameter. We used this approach to estimate the posterior marginal probability distributions for the parameters governing the influence of the host- and environmental-level, and spatial random effects predictors on the probability of infection. For each model we ran three independent Markov chains with varying initial values for 350,000 iterations and discarded the first 100,000. We thinned the Markov chains by keeping every twentieth iteration for inference. To determine if the three Markov chains used for each model had converged on the same posterior distribution, we used the statistical program R with the package boa [35] and employed several graphical and quantitative diagnostics, including autocorrelation plots, trace plots, and univariate corrected scale reduction factors for each parameter. To assess simultaneous convergence of all parameters for the top models, we calculated the multivariate potential scale reduction factor [32], [36], [37]. All inferences were based on the mean of each parameter’s marginal posterior distribution.

### Likelihood functions

The observation component of the data likelihood specifies each farm’s observed *M. bovis* infection status as a Bernoulli random variable with parameter 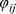:



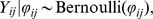
where Y*_ij_* is the infection status of the *i* ^th^ farm for *i*  =  1, …, *n* from the *j* ^th^ grid cell for *j*  =  1, …, *m*, and 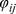 represents the probability of infection. Thus, given the probability of infection we assume each farm’s infection status is conditionally independent.

The process component of the data likelihood models, via the logit link function, defined as the probability of infection as a function of individual, environmental and spatial covariates as well as random effects that account for spatial variability:



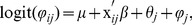
(1)where 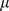 defines the baseline *M. bovis* infection probability, 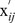 is the transpose of a 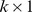 matrix of covariates for the *i* ^th^ farm from the *j* ^th^ grid cell, 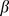 is a 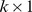 vector of parameter estimates for these covariates, 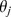 is a random effect for the *j* ^th^ grid cell capturing extra-binomial variability over the entire study region at the individual quarter township scale (HET; i.e., 25 km^2^), and 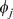 is a random effect term for the *j* ^th^ grid cell that models the extra-binomial variability associated with local disease clustering (CAR; i.e., grid cells closer together will have similar infection probabilities due to proximity of cattle farms and pastures).

### Prior distributions

We assumed non-informative *N* (0, 100,000) prior distributions for each of the 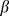 parameters, and an improper (flat) prior over the entire real line for *µ*. For the random effect describing region-wide heterogeneity (HET), we assumed the following:



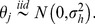
(2)


To describe the spatial structure, we assumed an intrinsic Gaussian conditional autoregressive prior with a sum to zero constraint [31] for the local clustering random effect (CAR):



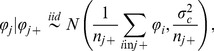
(3)where 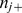 is the number of grid cells that share a border or vertex with the *j* ^th^ grid cell. Thus, the random effect of the *j* ^th^ grid cell is conditional on the values of its 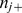 (usually  =  8) neighboring cells. Adjacency matrices were created with the Adjacency for WinBUGS Tool in ArcMap that provides a matrix relating one areal unit to a collection of neighboring areal units in text files for use in WinBUGS.

Because of the marginal specification for 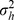 and conditional specification for 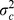 of the random effects, we generated prior distributions for the precisions (i.e., 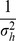 and 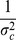) using simulations in WinBUGS where we varied the values of the parameters and determined the parameter values that created an expectation of ∼0.5 for the *psi* metric [38], where *psi* is as follows:



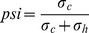
(4).

These simulations attempted to ensure an equal emphasis on the priors of the standard deviations of the random effects [31]. Based on our simulation results we defined


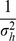
 ∼ Gamma(10.368,3.22) and 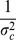 ∼ Gamma(1.0,1.0).

### Model Selection

To test our original hypothesis that environmental variables would influence the odds of *M. bovis* infection, our set of candidate models for logistic regression consisted of 12 different structures with strictly additive effects on the logit scale (Table 1). The 12 logistic regression models represented all possible combinations of deer and environmental variables, as well as inherent regional and local spatial structure of the data. Viewing these 12 models as competing hypotheses, we used deviance information criterion (DIC) [15], [33], [39] to compare the models’ respective fits to the data from sampled farms, and then estimated parameters and examined goodness-of-fit and other metrics for the top models. For model comparison we used DIC weights [33], which allow for an intuitive comparison of the evidence in the data for each candidate model. The weights are considered a measure of the strength of evidence in the data for *i* ^th^ model being the “best” model of those within the candidate set, and therefore provide a measure of model selection uncertainty [33], [39].

**Table 1:** Model selection results for the candidate set of models investigating the effect of covariates on the probability of bovine tuberculosis infection from 2005–2010 in Modified Accredited Zone in Michigan, USA using non-informative *N* (0, 0.00001) prior distributions for the fixed effects parameters and diffuse gamma priors for the random effects with farm-level factors removed.

Model Terms	Dbar	Dhat	pD	DIC	ΔDIC	Weights
Deer + ------ + HET + Envir	272.9	226.4	46.5	319.4	0.0	0.6386
Deer + ------ + HET + ------	276.2	231.5	44.7	320.9	1.5	0.3073
------ + ------ + HET + Envir	275.9	227.5	48.4	324.4	4.9	0.0540
Deer + CAR + ------ + ------	289.0	235.5	53.5	342.4	23.0	0.0000
------ + CAR + ------ + Envir	290.5	231.7	58.8	349.3	29.8	0.0000
Deer + CAR + ------ + Envir	298.1	232.0	66.1	364.2	44.7	0.0000
Deer + CAR + HET + ------	302.3	224.8	77.5	379.8	60.4	0.0000
------ + CAR + HET + Envir	309.3	226.2	83.1	392.3	72.9	0.0000
Deer + ------ + ------ + Envir	387.9	380.9	7.0	394.9	75.5	0.0000
Deer + ------ + ------ + ------	394.2	391.2	2.9	397.1	77.6	0.0000
------ + ------ + ------ + Envir	405.5	400.7	4.8	410.3	90.8	0.0000
Deer + CAR + HET + Envir	317.1	222.1	95.0	412.0	92.6	0.0000

“Deer” represents deer herd factors: apparent prevalence of deer and deer density. “Envir” represents the environmental variables: percent sand, percent ponding frequently, percent swamp/wetland, and mean soil pH in each sampled farms quarter township grid cell. “HET” represents the random effect capturing region-wide heterogeneity and “CAR” is the random effect capturing local clustering.

We used parameter estimates from the top model to calculate odds ratios for the effect of variables on *M. bovis* infection odds among farms in that area. Model averaging was not appropriate because DIC, unlike BIC and AIC, is not based on any assumption of a “true” model and is primarily concerned with short-term predictive ability [33]. We treated host-level (deer prevalence, deer density) and environmental-level predictors (percent sand, soil pH, proportion of wetland/swamp, and area that ponded frequently) as a group of variables, such that they were all entered or removed from the models together (Table 1). To examine the goodness-of-fit of the top model from our candidate set we conducted a numerical posterior predictive check [32]. We examined correlation and trace plots, as well as the estimates of the corrected scale reduction factor for each parameter and multivariate potential scale reduction factors and determined that that the three chains for each model had converged (data not shown). For each dataset, the top models selected via our model selection procedures and their corresponding estimates were similar regardless of prior specification (data not shown; [33]).

## Results

Out of the 762 cattle farms tested on an annual basis, 704 were negative while 37, 9, and 1 tested positive for *M. bovis* on 1, 2, and 3 occasions, respectively. Of our 12 models determined *a priori*, the top two models combined to account for over 95% of the summed weights of all models considered. Models weights of 95% provided strong assurance that some combination of these 2 models and their parameters reflected the underlying infection-generating process far better than other models in the candidate set (Table 1). Parameters in the top model included deer herd factors and local landscape features suggesting that these factors increased the odds of *M. bovis* infection to cattle in the northern, lower peninsula of Michigan (Table 2). As documented since initial diagnosis of a positive white-tailed deer in 1975, deer apparent prevalence ranged from 0.0% to 5.2% and was the most supported variable in the top model (odd ratio  =  1.004, 95% CI  =  1.001 to 1.007; Table 2). Sand within the vicinity of sampled farms was by far the most supported environmental variable and ranged from 37% to 79% on farms that test positive whereas sand ranged from 17% to 88% for cattle farms that tested negative for *M. bovis* (Fig. 1). The odds of infection for *M. bovis* increased by about 4% for every 1% increase in sand in the area (odd ratio  =  1.036, 95% CI  =  1.01 to 1.07; Table 2).

**Table 2:** Mean parameter estimates, standard deviation (SD), Monte Carlo error (MC error), odds ratios (OR), and 95% credible intervals for best-fitting model investigating the effect of covariates on the probability of bovine tuberculosis infection from 2005–2010 in Modified Accredited Zone in Michigan, USA.

Parameter	Mean	SD	MC error	2.50%	Median	97.5%	OR	95% CI
Intercept	−3.401	2.886	0.02	−9.219	−3.317	2.031	0.0333	0.00 to 7.622
Deer density	−0.2219	0.1454	0.00	−0.515	0.219	0.056	0.8001	0.60 to 1.06
Deer prevalence	0.4147	0.1412	0.00	0.137	0.414	0.697	1.004	1.001 to 1.007
Percent wetland	−0.0209	0.0262	0.00	−0.074	−0.020	0.029	0.9793	0.93 to 1.03
Percent sand	0.0357	0.0152	0.00	0.007	0.035	0.067	1.0363	1.01 to 1.07
Soil pH	0.04212	0.3368	0.00	−0.591	0.035	0.732	1.0430	0.55 to 2.08
Percent ponding	0.0240	0.035	0.00	−0.043	0.023	0.095	1.0243	0.96 to 1.10
HET	1.213	0.4819	0.00	0.524	1.128	2.39	3.3636	1.69 to 10.91

“HET” represents the random effect capturing region-wide.

Our analyses also identified that an unstructured random effect (HET) dominated over spatial structure (CAR) in influencing the odds of *M.bovis* infection (odd ratio  =  3.36, 95% CI  =  1.69 to 10.91; Table 2). Inclusion of the unstructured random effect in both our top models would suggest that additional covariates are driving odds of *M. bovis* infection and not spatial occurrence of *M. bovis*-positive farms in our study area.

## Discussion

Our findings support the premise that deer herd-related factors play an important role in sustaining *M. bovis* presence in the northern, lower peninsula of Michigan similar to that found in previous research [11], [40]. Because mitigation measures have been implemented to reduce deer access to feeding and cattle use areas [3], we focused our analysis simply on deer density and deer prevalence in the area without reference to farm practices. Farm practices have been the primary focus of most efforts to control *M. bovis* transmission between reservoirs and hosts [11], [18], [41], [42] but are difficult to standardize and document for inclusion in modeling efforts. Bayesian hierarchical models provide the ability to assess spatially the influence of region-wide cattle farm and host-level variables while adjusting for additional covariates [31], [43]. The bias that may have been introduced by entering 36% of cattle farms more than once into our models was deemed warranted to achieve our objectives of assessing environmental factors that may lead to continued presence of *M. bovis* on cattle farms. Furthermore, we don’t deny that farm-management practices are important in *M. bovis* infection on cattle farms, however, it is very difficult to accurately represent or measure one of these farm practices in a standardized format for hundreds of farms to include in our models. For this reason, we selected objectives that would look at determining new facet in understanding *M. bovis* infection (i.e., environmental variables) as opposed to conducting another study that suggested a different farm practice was responsible for bTB infection in cattle farms as documented in previous research [11], [18], [41], [42].

Although deer densities in the area have been reduced to about 10–15 deer/km^2^ since 1994 and deer prevalence has remained at just below 2% overall at the DMU 452-level [10], the role of a primary host for *M. bovis* in this region is still influencing transmission of *M. bovis* based on our study. Including mean deer prevalence for the duration of the study (i.e., 1998–2009) in our models would likely have yielded similar results to our current modeling effort but the role of the host would still be supported nonetheless. Due to public pressure about low deer densities and continued occurrence of cattle positive for *M. bovis* on an annual basis, reducing deer densities further is likely not possible. Relatedly, even with our conservative estimate of prevalence of *M. bovis* in deer (i.e., 2005–2009), deer prevalence was in the most supported model even though the odds ratio would likely have been greater if we included 1998–2009.

Our study identified environmental variables that were not possible to assess in previous modeling efforts that can further assist agencies in their attempts to eradicate *M. bovis* in Michigan. Environmental variables have been documented to contribute to presence or viability of infectious agents of disease in several areas in North American and Europe [17], [44], [45] although environmental sampling has yet to identify *M. bovis* on cattle farms in this region [46], [47]. Variables to consider should be based on *a priori* knowledge of the disease agent studied and mechanisms that may hinder or promote survival. Survival of *M. bovis* has been linked to moist, humid environments that maintain the proper soil type and pH [48]–[50]. Our top model indicated that a combination of landscape variables played an important role in determining infection probability for *M. bovis* on farms which was the impetus for us to select a combination of covariates that were conducive to moist, humid environments with low sunlight exposure on the landscape (e.g., wetlands, ponding frequency, soil types). Percent sand was a significant predictor and increased the odds of *M. bovis* infection by 3.6% for every 1% increase in proportion of sandy soil in the local area (Table 2). Survival of *M. bovis* has been linked to soil type, temperature and pH [3], [29], [48] but research in natural settings has limited the advancement of knowledge in this area.

The exact composition of sandy soils or functional role of these soils that make them conducive to the survival of *M. bovis* likely requires further research. Sandy soils are defined as having loose particle sizes (> 2 μm) with structural integrity during desiccation that may promote survival of *M. bovis* when associated with wetlands and areas that routinely have standing water. Moraines that have steep slopes and sandy, well-drained soils dominated by northern hardwood forests were linked to infection of white-tailed with *M. bovis* [51] but are confounded by the fact that they characterize preferred deer habitat in years with heavy oak mast production. Based on prevalence studies on white-tailed deer and our current study of farms positive for *M. bovis*, environmental or landscape-specific characteristics would appear to be a logical focus of future studies and potential assessment of viable *M. bovis* detection in soils or water. Areas that contain these sandy, well-drained soils in northern hardwood forests likely provide moist, humid microclimates conducive to survival of *M. bovis* for extended periods of time and should be considered for focused surveillance for *M. bovis* in deer and cattle as well as the focus of future on-farm mitigation measures.

Since initial detection of *M. bovis* in farms over a decade ago, farms have tested positive on an annual basis. Even after considerable efforts have been implemented to reduce deer densities, limiting or preventing aggregation of deer (e.g., ban on baiting of deer), and limiting deer-cattle interactions through on-farm mitigation measures, Michigan still does not have *M. bovis*-free status. Although modeling efforts are unable to include movements of cattle between farms and its influence on movements and spread of *M. bovis*, repeated positive tests have occurred on numerous farms since the first farm tested positive in 1998 [28]. Repeated positive tests would suggest that potentially an environmental source conducive to survival of the bacteria may be responsible for maintaining *M. bovis* in the region. Mycobacteria have waxy, lipid-rich cell walls that are relatively resistant to biocides used in decontamination procedures thus complicating management of the disease.

Unlike previous work in this region, we were able to assess spatial processes that may be influencing the transmission or presence of *M. bovis* using a Bayesian hierarchical modeling framework [31], [43]. We identified that unstructured spatial heterogeneity (HET) was included in the top two models explaining infection of *M. bovis* on farms. If probability of infection was driven by spatial structure or clustering of disease (i.e., contiguous grid cells more alike than 2 arbitrary grid cells) at our site, we would have expected spatial structure (CAR) to be included in our top models but the opposite occurred [43]. Unstructured spatial heterogeneity would suggest that additional covariates that we may not have accounted for in our models were also influencing the disease across our study region. As stated previously, movement of cattle between farms and additional on-farm practices is controlled or mitigated to some extent for some farms but is very difficult to enforce and has been documented to be the cause of contamination of several cattle farms within and outside of the MAZ [21]. Our unstructured spatial heterogeneity could simply be on-farm practices not included in our modeling effort or additional environmental covariates conducive to survival or destruction of *M. bovis* such as slope/aspects conducive to direct exposure to ultraviolet light.

Although host prevalence in DMU 452 and clustering of cattle farms were considered important in *M. bovis* infection in previous research [11], [52], our results suggest that less spatially structured components in the landscape are influencing continued occurrence of *M. bovis* in cattle on farms in Michigan. Considering the logistics of managing the host and reservoir through the northern, lower peninsula of Michigan, management efforts could focus on environmental and landscape characteristics that are potentially supporting the continued presence of *M. bovis* in Michigan. Landscape characteristics that are conducive to survival of *M. bovis* such as habitat inundated with standing water, soil composition, and prime deer habitat should be the focus of future management and research. On-farm mitigations should focus efforts in areas at high risk for continued survival of *M. bovis* prior to mandating complete risk mitigation of farms for an entire area such as DMU 452 (1,479 km^2^) or the 5 county study area (8,062 km^2^). Focused surveillance and management of on-farm practices in reservoirs for disease in domestic livestock would provide a logistically feasible approach to combating a disease in an endemic area as well as new areas that the disease has recently been introduced or spread from the endemic area.
